# Myocardium Detection by Deep SSAE Feature and Within-Class Neighborhood Preserved Support Vector Classifier and Regressor

**DOI:** 10.3390/s19081766

**Published:** 2019-04-13

**Authors:** Yanmin Niu, Lan Qin, Xuchu Wang

**Affiliations:** 1 Key Laboratory of Optoelectronic Technology and Systems of Ministry of Education, College of Optoelectronic Engineering, Chongqing University, Chongqing 400044, China; qinlan@cqu.edu.cn (L.Q.); xcwang@cqu.edu.cn (X.W.); 2 College of Computer and Information Science, Chongqing Normal University, Chongqing 400050, China

**Keywords:** myocardium detection, cardiac magnetic resonance, region proposal, support vector classifier and regressor, stacked sparse autoencoder (SSAE)

## Abstract

Automatic detection of left ventricle myocardium is essential to subsequent cardiac image registration and tissue segmentation. However, it is considered challenging mainly because of the complex and varying shape of the myocardium and surrounding tissues across slices and phases. In this study, a hybrid model is proposed to detect myocardium in cardiac magnetic resonance (MR) images combining region proposal and deep feature classification and regression. The model firstly generates candidate regions using new structural similarity-enhanced supervoxel over-segmentation plus hierarchical clustering. Then it adopts a deep stacked sparse autoencoder (SSAE) network to learn the discriminative deep feature to represent the regions. Finally, the features are fed to train a novel nonlinear within-class neighborhood preserved soft margin support vector (*C*-SVC) classifier and multiple-output support vector (ε-SVR) regressor for refining the location of myocardium. To improve the stability and generalization, the model also takes hard negative sample mining strategy to fine-tune the SSAE and the classifier. The proposed model with impacts of different components were extensively evaluated and compared to related methods on public cardiac data set. Experimental results verified the effectiveness of proposed integrated components, and demonstrated that it was robust in myocardium localization and outperformed the state-of-the-art methods in terms of typical metrics. This study would be beneficial in some cardiac image processing such as region-of-interest cropping and left ventricle volume measurement.

## 1. Introduction

Cardiovascular diseases (CVDs) remain the leading cause of death and disability globally. For years, a great effort has been dedicated to the prevention, diagnosis, treatment and research of CVDs. The hardware and software developments have been helping the increasing use of cardiovascular magnetic resonance imaging (MRI) in this effort. It is essential to detect the important structures of a left ventricle myocardium from MRI scans in a clinical-decision support system dedicated to improving the early diagnosis of critical CVD diseases. For example, accurate myocardium location will be very helpful for subsequent processing such as cardiac image registration and tissue segmentation, also for understanding cardiac anatomy how to adapts to disease [1]. Computer-aided automatic detection provides great potential to solve this problem instead of tedious, time-consuming, and poorly reproducible manual detection. However, this has been a challenging task due to the complex structure of cardiac anatomy, and low image quality such as presence of noise, low contrast and intensity non-uniformity [2,3,4].

### 1.1. Related Works

Myocardium detection is a task that has benefited from the object detection in the computer vision field. Traditionally, hand crafted features, such as HOG (histogram of oriented gradients), SIFT (scale-invariant feature transform), Haar-like feature, etc, are widely used to train various classifiers [5]. The enhanced cascade detector [6] that was originally developed for face detection, and the decision forest detector that combines a wide range of contextual characteristics and random forest classifier to locate nine different organizations on human body images [7].

However, along with recent breakthrough works in deep learning field, many CNN (Convolutional Neural Network) architectures have been studied for object detection [8,9,10,11,12] and achieved more satisfactory performance in nature or optical images. Typically, these works can be roughly divided into two categories: region proposal based methods and region proposal free methods. The former mainly includes RCNN (Regional Convolutional Neural Network) [8], SPP-Net (Spatial Pyramid Pooling Net) [13], Fast-RCNN [14], Faster-RCNN [9], R-FCN (Region-based Fully Convolutional Network) [15] and its multi-scale version [16] and cascaded improvement [17]. Furthermore, the relation among the detected objects is modeled by a CNN network with two full connected layers [18], and the iterative localization refinement is designed to facilitate object localization by undertaking at a mid-layer of a CNN to progressively refines a subset of region proposals [19]. The second category is not using the region proposal, such as three versions of YOLO (You Only Look Once) [10,20,21] and SSD (Single Shot MultiBox Detector) [22] and its improvement [23].

In this work, we focus on region proposal-based methods since they are applied by most of the top-performing object detection methods. In this two-stage method, a sparse set of candidate regions is first generated, and then they are further classified and regressed. The representative RCNN built the relationship between image classification and object detection by three steps: First, selective search [24] is applied to generate around 2000 category-independent region proposals in stead of the traditional sliding window approach. Second, the features of each region proposal are extracted by a pretrained CNN model. Third, the top-level features are classified by linear SVM (Support Vector Machines). RCNN has a solid pipeline but its computation speed is slow because it performs a CNN forward pass for each object proposal, without sharing computation. Fast RCNN [14] combines the region proposal classification and bounding box regression tasks into one single stage to speed up the detection. Moreover, the region of interest pooling strategy based on the top-level features is more efficient than the RCNN feature extracting method. In other words, multi-task training avoids managing a pipeline of sequentially-trained tasks. Nevertheless, because selective search is applied to generate region proposals in Fast RCNN, thereafter the detection speed of Fast RCNN is affected. Faster RCNN [9] solves the proposal computation bottleneck of Fast RCNN by using a region proposal network that is a kind of fully convolutional network and can be trained end-to-end to generate detection proposals.

Generally, these works mainly aim to improve the object detection accuracies in two ways: (1) optimizing the architecture of the CNN, take full advantages of the distinguished ability of the deep feature learned by CNN; (2) exploring how to share computation among different proposed regions, which will speed up the whole detection process. Along with these strategies, some deep learning techniques have been applied for medical object detection, for example, Yan et al. [25] uses a system containing two convolutions depth convolution neural network, with 7000 two-dimensional axis of the slice image training, and ultimately to the body of 12 different organizations. Vos et al. [26] trained three independent CNNs based ROI detectors, where each classified 2D image slices from one of three orthogonal image planes (axial, sagittal, or coronal), then all of them were combined to determine a rectangular 3D bounding of anatomical ROI. This method achieved good detection results, but because of the need to train multiple networks, the algorithm itself was less efficient. Roth et al. [27] trained a 5-layer convolution neural network by using 4300 two-dimensional axial-shaped slices on the human body of five different regions (legs, pelvic, liver, lung, and neck). In the application to cardiac tissue detection, Luo et al. [28] employed a 8-layer fully convolutional networks to locate the ROI (Region Of Interest) that contains the bi-ventricular regions for right ventricle segmentation. Poudel et al. [29] proposed a recurrent fully-convolutional network that combines left ventricle detection and segmentation into a single architecture that is trained end-to-end thus simplifying the segmentation pipeline. Tan et al. [30] proposed a CNN network with fully connected layer to regress the location of left ventricle center point in cardiac images. Vigneault et al. [4] built a small localization network from the layer immediately following the final max pooling of U-Net to predict the transformation parameters in locating the left ventricle in cardiac images. This method can output the directed localization but contains much more surrounding tissues. Overall speaking, the investigation on myocardium detection is very limited in comparison to the advances in natural object detection and most existing approaches usually take it as a module in the pipeline of cardiac segmentation, where they strengthen the completeness of myocardium more remarkably than the accuracy.

### 1.2. Motivation and Contribution

In above methods, Faster RCNN obtains both high detection efficiency and detection accuracy without changing the pipeline of region proposal and region classification. Unfortunately, four problems are not solved in the studies. Firstly, the network training requires huge amount of labeled training data, which makes it hard for medical image applications to utilize this technology, due to the fact that it is extremely hard to collect such a large data with correctly diagnosed labels. Secondly, contextual information is not integrated with the top-level features. Thus, the quality of generated region proposals is relatively poor. Thirdly, the design for the selected scales and aspect ratios of anchor boxes is not optimal for medical objects because in our task there is some anatomical constraints that cannot be described as the limited scales and aspect ratios. Therefore, the ability of regional proposal network object localization is weak for myocardium detection. Fourthly, the classifier is not optimal for solving binary classification problem since it does not consider the structure information of the top-level features. As a result, the performance is affected in detection of specific medical tissues.

The objective we consider is to localize myocardium and left ventricle tissue (or, left ventricle ROI) in cardiac MRI images, where the object is single and the anatomical information is more remarkable in comparison to multiple objects in natural images. However, the localization remains a challenging problem due both to intrinsic and extrinsic difficulties. Intrinsic difficulties refer to the essential properties of the MR imaging systems that result in imaging noise and the complexity of cardiac tissues. The major source of noise that degrades image quality is mainly caused by radiation scattering and source leakage. The complexity of cardiac tissues lies on that in typical short-axis steady-state free precession cine MR images, the contrast between the blood pool within the left ventricle and the endocardial wall is varying, the interference of endocardial trabeculation and papillary muscles is relative strong, and the contrast between the epicardial wall and surrounding structures is extremely weak and varying, particularly against low-signal lung tissue. Extrinsic difficulties are closely related to the patients with biological variability in heart size, orientation in the thorax, and morphology across subjects. Also there is variability in contrast and image appearance with different scanners, protocols, and clinical planes. In some images, the borders between the ventricles and the atria, and the separation between the chambers and the vasculature are hard to define. In this context, it is admirable to generate candidate regions according to the texture and structure information of the cardiac image, instead of the convolution network-based region proposal network. Also a stronger classifier is necessarily investigated to distinguish the proper proposal regions from large mount of candidates.

Motivated by these observations, in this paper, we propose a hybrid model to detect myocardium by integrating region proposal and deep feature classification and regression. Our model firstly generates candidate regions on cardiac images using new structural similarity-enhanced supervoxel over-segmentation plus hierarchical clustering. Compared with the typical sliding window extraction methods, the algorithm is more efficient and generates less redundant regions. Then it adopts a deep SSAE (Stacked Sparse Auto-Encoder) network to learn the deep feature to represent the candidate regions. Compared with the traditional manual feature, the difference between the myocardium region and background is strengthened by the supervised SSAE deep learning, which also improves the robustness of our model. Finally, the obtained deep feature represented candidate regions are fed to train a nonlinear within-class neighborhood preserved soft margin support vector classifier (*C*-SVC) and multiple output support vector regressor (ε-SVR), and finally output the myocardium region. To improve the stability and generalization, the proposed model also takes the hard negative sample mining strategy to fine-tune the SSAE and the classifier. Experimental results show that the accurate myocardium detection results can be achieved by fine-tuning a pre-trained deep learning network.

Briefly speaking, the contributions and advantages of our method are highlighted as follows:

(1) The region proposal is generated using new structural similarity-enhanced supervoxel over-segmentation plus hierarchical clustering. Instead of the color and spatial similarity computation in the widely adopted SLIC (Simple Linear Iterative Clustering) method, the proposed supervoxel introduced a sequence of measure, including image phase congruency, intensity, contrast, structure, and coordinates to enhance the structural similarity, which can easily take into account the context of similar anatomical tissues while limiting the capacity of redundant proposals.

(2) The SSAE network is introduced to learn the deep features related to region proposals. SSAE enjoys all the benefits of any deep network of greater expressive power by capturing a useful “hierarchical grouping” or “part-whole decomposition” of the candidate regions. For supervised SSAE, the gradients from the softmax classification error will then be back-propagated into the encoding layers and which can enhance the difference of feature representation for positive and negative regions. Furthermore, SSAE outputs less feature by dimensionality reduction to help training more robust classifier and regressor in the successive steps.

(3) The nonlinear within-class neighborhood preserved *C*-SVC classifier and ε-SVR regressor are proposed to classify the SSAE-learned features and regress the locations of features to refined positions, respectively. Since there are common components in many features due to their intensity representation of limited anatomical types of cardiac images, these components will be encoded as more similar parts after SSAE learning. According to this, we propose a nonlinear regularization to preserve the within-class neighborhood structure and incorporate it to *C*-SVM and ε-SVR. It can alleviate the limitations that standard *C*-SVM and ε-SVR suffer from the noisy data that heavily affect the hyperplane, since they obtain larger non-zero coefficients after training. In addition, our multiple-input multiple-output ε-SVR can produce more robust nonlinear regression than the linear regression in many region-based detectors.

We intensively investigated the performance of the proposed method with impacts of different components and compared it with related methods on public available cardiac data set. Although Faster RCNN has become one of the most outstanding detection methods for natural images, we show that the proposed method can achieve competitive results more efficiently and has potential as well when incorporating the integration of enhanced supervoxel-based region proposal, deep learned SSAE feature, and nonlinear within-class neighborhood preserved *C*-SVC classifier and ε-SVR regressor. In this context, the proposed model is valuable as a reference for segmenting other similar medical objects in limited image sets.

The rest of the paper is organized as follows. Section 2 describes the flowchart of proposed model and the details of major modules. Section 3 presents the data set and our evaluation metrics. Experimental results and discussion are reported in Section 4 and we summarize our work in Section 5.

## 2. Proposed Method

### 2.1. Overview of Our Detection Model

**Figure 1:**
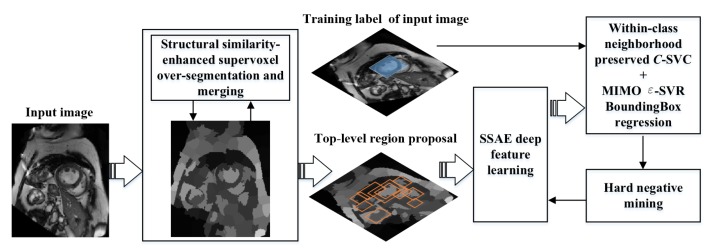
Training flowchart of proposed myocardium detection model.

We formulate the myocardium detection as a classification and regression problem and the training flowchart of our model is shown in Figure 1. This model consists of four modules: (1) candidate region extraction module that combines structural similarity-enhanced supervoxel algorithm and hierarchical clustering to generate target candidate regions; (2) feature learning module that extracts the deep SSAE characteristics of the candidate regions; (3) region location module that trains a within-class neighborhood preserved *C*-SVC classifier to determine and locate the myocardium region; (4) refinement module that collects hard-to-be-classified samples and use them to fine tune the model, also eliminates redundant (cross-repeated) candidate regions and find the best target detection position by using the NMS (Non-Maximum Suppression) and ε-SVR bounding box regression strategies. In the following subsections, we will present the details of each module.

### 2.2. Candidate Region Proposal

In two-stage object detection methods, it is usual to generate the candidates in possible sizes, scales, locations to handle the strong randomness of target located on the image. The first early region candidate generation algorithm adopted sliding window [6] to traverse the entire images, the image needs to be set as different scales and sliding window aspect ratio. This exhaustive search certainly could find the target, meanwhile it generated more redundant proposals with the much high time complexity, furthermore, the huge negative proposals makes the classifier less sensitive to the positive proposals. To overcome the limitation, selective search [24], edge boxes [31], region proposal network [9], superpixel proposal [32,33,34] were introduced to generate candidate regions quickly and efficiently. For our detection task, there are many similar anatomical structures in the limited cardiac images, so we consider the supervoxel-based over-segmentation to generate the initial regions.

#### 2.2.1. Structural Similarity-Enhanced Supervoxel Over-Segmentation

The distance or similarity measure plays an essential role in supervoxel framwork. For typical SLIC, the feature for distance measure is built as LAB-color space-based intensities balanced with the pixel location distance [33,35]. No structural information is incorporated. For cardiac image, this may be less appropriate since the intensity is single channel and objects are usually with coarse boundaries. So we introduce five parts to enhance the structural similarity as follows.

(1) Phase congruency measure

Image phase congruency (PhaseCong) model assumes the visual feature should be high in information (or entropy), and low in redundancy. Instead of searching for points where there are sharp changes in intensity, this model searches for patterns of order in the phase component of the Fourier transform. Based on the physiological and psychophysical evidences, the PhaseCong theory provides a simple but biologically plausible model of how mammalian visual systems detect and identify features in an image. Rather than define features directly at points with sharp changes in intensity, the PhaseCong model postulates that features are perceived at points where the Fourier components are maximal in phase. PhaseCong can be considered as a dimensionless measure for the significance of a local structure [36].

To compute the PhaseCong of cardiac images, we can apply the two-dimensional log-Gabor filters that uses the Gaussian spreading function across the filter perpendicular to its orientation. In this way, the phase of any function would stay unaffected after being smoothed with Gaussian. Thus, the phase congruency would be preserved. This function has the following transfer formulation

(1) $$G\left(\omega ,\theta \right)=\exp\left(-\frac{log{\left(\omega /\omega_{0}\right)}^{2}}{2\sigma_{r}^{2}}\right)\exp\left(-\frac{{\left(\theta -\theta_{j}\right)}^{2}}{2\sigma_{\theta }^{2}}\right),$$

where $\omega_{0}$ is the filter’s center frequency and $\sigma_{r}$ controls the filter’s bandwidth; $\theta_{j}$ is the orientation angle of the angle filter and $\sigma_{\theta }$ determines the filter’s angular bandwidth. By modulating $\omega_{0}$ and $\theta_{j}$ and convolving *G* with the image, a set of responses at each point *u* as $\left[e_{n,\theta_{j}}\left(u\right),o_{n,\theta_{j}}\left(u\right)\right]$. The local amplitude on scale *n* and orientation $\theta_{j}$ is $A_{n,\theta_{j}}\left(u\right)=\sqrt{{\left(e_{n,\theta_{j}}\left(u\right)\right)}^{2}+{\left(o_{n,\theta_{j}}\left(u\right)\right)}^{2}}$ and the local energy along orientation $\theta_{j}$ is $E_{n,\theta_{j}}\left(u\right)=\sqrt{{\left(\sum_{n}e_{n,\theta_{j}}\left(u\right)\right)}^{2}+{\left(\sum_{n}o_{n,\theta_{j}}\left(u\right)\right)}^{2}}$, therefore, the phase congruency at the point *u* is obtained as

(2) $$PhaseCong\left(u\right)=\frac{\sum_{j}E_{\theta_{j}}\left(u\right)}{\sum_{n}\sum_{j}A_{n,\theta_{j}}\left(u\right)},$$

and the phase congruency measure of two points are defined as

(3) $$S_{pm}\left(u,v\right)=\frac{2PhaseCong\left(u\right)PhaseCong\left(v\right)+c_{1}}{PhaseCong{\left(u\right)}^{2}+PhaseCong{\left(v\right)}^{2}+c_{1}}.$$

(2) Intensity measure

For two patches Pat(u) and Pat(v) centered at position *u* and *v*, with d=p×p patch size in cardiac image (p=7or9 is better in our study), we define the intensity measure as $S_{mm}\left(u,v\right)=\frac{2\mu_{u}\mu_{v}+c_{2}}{\mu_{u}^{2}+\mu_{v}^{2}+c_{2}}$, where $\mu_{u}=1/d\sum_{i=1}^{d}Pat_{i}\left(u\right)$ and $\mu_{v}=1/d\sum_{i=1}^{d}Pat_{i}\left(v\right)$ are the mean intensities of the compared patches, and constant $c_{2}$ is included to avoid instability when the intensities of two patches are near to zero. If the mean intensities of two patches are close, $S_{mm}\left(u,v\right)$ will approach to 1 and vice versa.

(3) Contrast measure

Once the mean intensity is removed from each patch, the resulting signal can be seen as the inner contrast of the patches, so we use the standard deviation to estimate the similarity of these contrast, i.e., $S_{cm}\left(u,v\right)=\frac{2\sigma_{u}\sigma_{v}+c_{3}}{\sigma_{u}^{2}+\sigma_{v}^{2}+c_{3}}$, where $\sigma_{u}^{2}=1/\left(d-1\right)\sum_{i=1}^{d}{\left(Pat_{i}\left(u\right)-\mu_{x}\right)}^{2}$ and $\sigma_{v}^{2}=1/\left(d-1\right)\sum_{i=1}^{d}{\left(Pat_{i}\left(v\right)-\mu_{v}\right)}^{2}$, $c_{3}$ plays the same role as $c_{2}$. If the mean contrast of two patches are close, $S_{cm}\left(u,v\right)$ will approach to 1 and vice versa. Furthermore, this measure is less sensitive to the case of high base contrast than low base contrast and consistent with the contrast-masking feature of the human visual system.

(4) Structure measure

For two patches Pat(u), Pat(v) centered at *u* and *v*, correlation (inner product) between them is a simple but effective measure to quantify their structural similarity. Since it equals to the correlation coefficient of the normalized patches with voxels $\left(Pat_{i}\left(u\right)-\mu_{u}\right)/\sigma_{u}$ and $\left(Pat_{i}\left(v\right)-\mu_{v}\right)/\sigma_{v}$, so we define the structure measure as $S_{sm}\left(u,v\right)=\frac{2\sigma_{uv}+c_{4}}{\sigma_{u}\sigma_{v}+c_{4}}$, where $\sigma_{uv}=1/\left(n-1\right)\sum_{i=1}^{d}\left(Pat_{i}\left(u\right)-\mu_{u}\right)\left(Pat_{i}\left(v\right)-\mu_{v}\right)$. Geometrically, the correlation coefficient corresponds to the cosine of the angle between the vectors with elements $\left(Pat_{i}\left(u\right)-\mu_{u}\right)$ and $\left(Pat_{i}\left(v\right)-\mu_{v}\right)$, so we take the absolute operation to constrain it into the range of 0 and 1.

(5) Coordinate measure

The coordinate measure is defined as $S_{dm}\left(u,v\right)={\exp(-\alpha ||u-v||}^{2})$, where α is related to the supervoxel number *K* and image voxel number *N*. Typically, $\alpha =2\sqrt{K/N}$. Different from the distance measure in SLIC method, this definition explicitly constrains the similarity into [0,1].

Then, these measurements are combined to get the hybrid similarity of patches centered at the position *u* and *v* on the image. We define S(u,v) as

(4) $$\begin{array}{cc} S(u,v)= & {\left[S_{pm}\left(u,v\right)\right]}^{\beta_{1}}{\left[S_{im}\left(u,v\right)\right]}^{\beta_{2}}\\ & {\left[S_{cm}\left(u,v\right)\right]}^{\beta_{3}}{\left[S_{sm}\left(u,v\right)\right]}^{\beta_{4}}{\left[S_{dm}\left(u,v\right)\right]}^{\beta_{5}}, \end{array}$$

where $\beta_{i}\left(i=1,\cdots ,5\right)$ are weights for the corresponding part, in our experiments, we let them as 1 for simplicity. This comprehensive distance measure explicitly integrates the different measures of two patches into a unified measure space, and it can be separately calculated and then incorporated to form the final results for speeding up the computation.

#### 2.2.2. Supervoxel Region Merging by Hierarchical Clustering

The initial over-segmentation regions by the supervoxel algorithm usually divide the objects into many adjoint parts. These regions should be adjusted to represent the object more accurately and efficiently. We introduce hierarchical clustering algorithm to merge the generated regions in a bottom-up way. Each of the two merged regions satisfies two conditions: (1) the regions should be adjacent; (2) the two regions have the highest similarity. The similarity is computed using Equation (4), but in a supervoxel region instead of patch, and the coordinate measure are computed in the center of the supervoxel instead of locations of voxels. Theoretically, the final result of clustering is that all the initial regions are merged into the same region (that is, the whole image). So, it is necessary to set the desired number of final regions, so that a candidate target area for detection can be finally obtained.

The complete candidate region generation algorithm is described as follows:

Step 1: Generating initial over-segmentation regions by supervoxels framework with the similarity measure in Equation (4) in a patch-wise way;

Step 2: Calculating similarity between all adjacent regions by using the similarity measure in Equation (4) in a supervoxel-wise way;

Step 3: Sorting the similarities, and then merging the two regions with the highest similarity to form new over-segmentation regions;

Step 4: Repeat Steps 2 and 3 until the number of remaining areas reaches to the predefined value.

**Figure 2:**
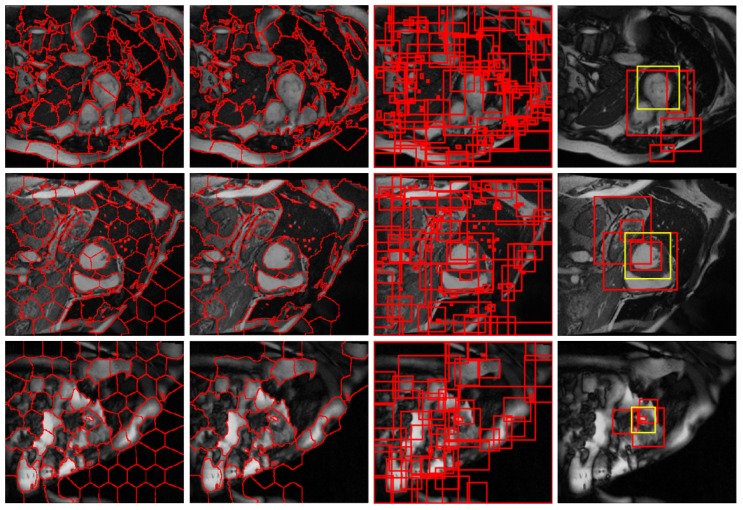
Procedure of our region proposal generation. From left to right: initial structural similarity-enhanced supervoxels; merged supervoxels by hierarchical clustering; corresponding bounding boxes; final top-level region proposals where yellow rectangles denote ground truth.

Figure 2 illustrates the procedure of our region proposal generation on three typical images located in the base, middle, and apex parts of myocardium tissue, respectively. It is seen that the targets both include in-homogeneous blood pools and myocardium and show much variability in complex background. The endocardium is not always closed circle while the epicardium exhibits obscure boundaries to surrounding tissues. Our initial over-segmentation extract the anatomical structures in most cases, but still separate the targets into different adjacent parts. After hierarchical clustering this disjoint limitation is greatly decreased, and the final region proposals covers the true objects through some restrictions, e.g., the ratio of height and width in bounding box, the number of voxels in supervoxels, the location near to the image border. It is also noticeable that our approach generates much less proposal than selective search-based methods.

### 2.3. Deep SSAE Feature Learning

It is difficult to design a hand-crafted feature to capture the characteristics of left ventricle and myocardium tissue in different cardiac images because the gray scales of left ventricle target in heart MRI images are varying in complex morphological changes and image background. For learning-based detection model, an effective feature representation can relieve this burden. In view of the excellent ability and robustness of the deep learning characteristics, this paper propose to extract deep feature representation of the proposal regions using deep SSAE model.

To be specific, the region proposal generation algorithm outputs candidates with different scales and different sizes according to supervoxel and regional hierarchical clustering. For each candidate, the minimum bounding rectangle is built and the corresponding region from the original image is cut down to form a training sample. Since the dimensions of bounding rectangles are different across the images, they should be scaled to a fixed size (i.e., τ×τ ). For SSAE, the number of neurons in the input layer is the dimension of the training sample, so the value of $\tau^{2}$ is directly determined by the number of neurons in the SSAE input layer, which is one of the important parameters of SSAE structure. In our experiments, the value of τ is settled via cross-validation.

**Figure 3:**
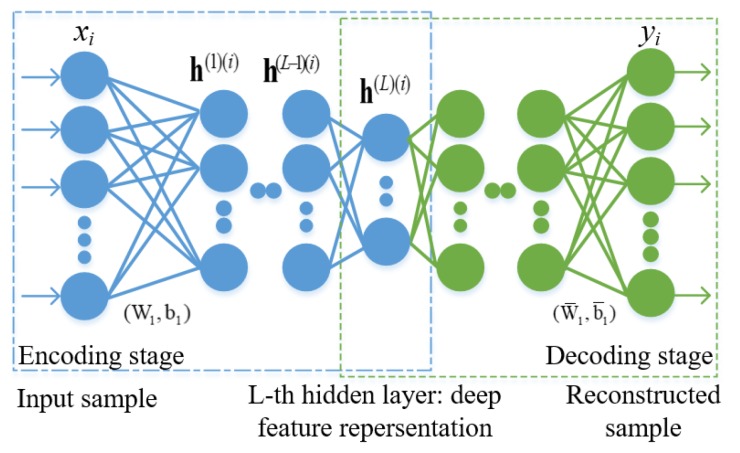
SSAE structure with three hidden layers in this work.

SSAE is a deep neural network composed of multiple stacked sparse auto-encoders (SAEs) [37], and it has been applied in tissue segmentation in late gadolinium-enhanced cardiac MRI images (such as atrial scarring segmentation [38], atrial fibrosis segmentation [39], left atrium segmentation [40]), nuclei patch classification on breast cancer histopathology images [41], brain tissue segmentation in visible human images [42], or other applications (such as hyperspectral imagery classification [43] and building extraction from LiDAR and optical images [44]). Figure 3 shows a SSAE network with three hidden layers, where a SAE aims to learn features that form a good sparse representation of its input. The first layer of a SSAE tends to learn first-order features in the raw input (such as edges in a proposal). The second layer tends to learn second-order features corresponding to patterns in the appearance of first-order features (e.g., in terms of what edges tend to occur together). Higher layers of the SSAE tend to learn the sparse but even higher-order features, which can be admirable for classifying myocardium regions.

SSAE includes encoding and decoding phases and each phase contains more layers (here, three, which can be set according to specific tasks). Given an input sample *x*, the first SAE maps it to the activation vector $h^{\left(1)(i\right)}\left(x_{i}\right)=f\left(W_{1}x_{i}+b_{1}\right)$, where f(z)=1/(1+exp(-z)) is the sigmoid function to make non-linear activation, $W_{1}\in R^{H\times M}$ represents the coding weight matrix in the first layer of the sparse self-encoder, $b_{1}$ represents the offset variable; then, this vector $h^{\left(1)(i\right)}\left(x_{i}\right)$ is used as the input vector for the second SAE mapped to activation vector $h^{\left(2)(i\right)}\left(x_{i}\right)$; in a similar way, this vector is finally expressed as the final depth characteristic of the input sample. The average hidden layer activation value of this neuron for all training samples can be expressed as ${\hat{\rho }}_{j}=\frac{1}{n}\sum_{i=1}^{n}h_{j}\left(x^{\left(i\right)}\right)$, in this condition, we can set a small sparse parameter (e.g., ρ=0.01), by making ${\hat{\rho }}_{j}=\rho$, the mean activation of each neuron will be close to ρ, so as to achieve the purpose of sparse constraints and it can be formulated as

(5) $$\sum_{j=1}^{s_{2}}KL(\rho ||{\hat{\rho }}_{j})=\sum_{j=1}^{s_{2}}\rho \log\frac{\rho }{{\hat{\rho }}_{j}}+\left(1-\rho \right)\log\frac{1-\rho }{1-{\hat{\rho }}_{j}}.$$

Here, $s_{2}$ represents the number of neurons in the implicit layer, and the index *j* represents the *j*th hidden layer neurons. $KL(\rho ||{\hat{\rho }}_{j})$ is a Kullback-Leibler divergence that measures the similarity between two different distributions ρ and ${\hat{\rho }}_{j}$.

The overall cost function of SSAE is defined as

(6) $$J=\frac{1}{n}\sum_{i=1}^{n}\left(\frac{1}{2}||y^{\left(i\right)}-x^{\left(i\right)}|{|}^{2}\right)+\lambda \frac{1}{2}\sum_{l=1}^{p-1}\sum_{i=1}^{s_{l}}\sum_{j=1}^{s_{l-1}}{\left(W_{ji}^{\left(l\right)}\right)}^{2}+\beta \sum_{j=1}^{s_{2}}KL(\rho ||{\hat{\rho }}_{j}),$$

where the first term is defined as a mean square error cost function to learn an identity function so that output $y_{i}$ equals to input $x_{i}$. The second one is a quadratic regularized function to penalize the parameters *W* and reduce the risk of the model being over-fitted. The third term is a sparse constraint.

The training of SSAE is the process of optimizing its cost function. It is generally conducted using step-by-step greedy strategy. It consists of two parts: (1) model pre-training (progressive training for each SAE); (2) model fine-tuning (fine-tuning of the pre-trained model). In the first stage, the training samples are used to train the first layer of SAE separately. After this training is completed, the second layer SAE is trained by using the hidden layer output activation vector of the first layer SAE, and then the output activation vector of the second layer SAE is used to the training of the third layer of SAE (if there are more hidden layers, the training processes in the same way). After the first part of the training is completed, a number of trained SAE is stacked into a multi-layer SSAE, the initial parameters of SSAE are feed by the weight parameters of SAE, and the last hidden layer of neurons connects to the classifier to form a complete classification network. The gradient descent algorithm is used to pass the error of the Softmax classifier to the entire network, and the whole network is then fine-tuned [37,45].

### 2.4. Within-Class Neighborhood Preserved *C*-SVC Classification

In this paper, the two-class soft margin support vector classifier is considered to classify the candidate regions (each candidate region is divided into a target area or a non-target area). Standard *C*-SVM suffers from the noisy data that heavily affect the hyperplane, since they obtain larger non-zero coefficients after training [46]. In addition, the feature learned by SSAE is biased for the negative samples, that are the mixture of the complex background. To alleviate these limitations, we propose to incorporate local geometric structure to constrain the maximum margin-based *C*-SVC, and the classifier is built as follows.

Suppose there are *N* SSAE learned samples $x_{i}\left(i=1,\cdots ,N\right)$ and they belong to class $C_{k}\left(k=1,2\right)$, and the size of each class is $N_{k}$. There is a linear or nonlinear mapping ϕ to transform x into an arbitrary reproducing kernel Hilbert space H, i.e., $\phi :R^{D}\mapsto H$, then, according to the Mercer theorem, a kernel function $K\left(x_{i},x_{j}\right)=\phi {\left(x_{i}\right)}^{T}\phi \left(x_{j}\right)$ could be designed to avoid curse of dimensionality. Exploiting the manifold in the form of a graph can be seen as a method of incorporating local proximity information of the images into the dimensionality reduction framework, that can enhance the clustering quality in the low-dimensional space. Assume the mapped data are centered in H, i.e., $\sum_{i=1}^{N}\phi \left(x_{i}\right)=0$, and total scatter matrix of samples in feature space is $S_{t}^{\phi }=\frac{1}{N}\sum_{i=1}^{N}\phi \left(x_{i}\right)\phi {\left(x_{i}\right)}^{T}$, the eigen-decomposition is $S_{t}^{\phi }v=\lambda v$; $v\in span\{\phi \left(x_{1}\right),\phi \left(x_{2}\right),\cdots ,\phi \left(x_{N}\right)\}$. Let $v=\sum_{i=1}^{N}\mu_{i}\phi \left(x_{i}\right)$, we obtain λNμ=Kμ due to K is symmetric and has a set of eigenvectors spanning the whole space. In case that the mapped data are not centered in H, we replace K by $\left(I-ee^{T}\right)K\left(I-ee^{T}\right)$ to implement centralization, where $e=N^{-1/2}1_{N}^{T}$. In this way, a sample $\phi (x_{i})$ obtains its projection as

(7) $$\phi {\left(x_{i}\right)}_{kpca}=W_{kpca}^{T}K\left(x_{i},\cdot \right),$$

where $W_{kpca}=\left[\mu_{1},\mu_{2},\cdots ,\mu_{N-1}\right]$ with arrangement of $\mu_{i}$ according to the descending order of eigenvalues. This KPCA preprocessing does not lose any information due to the representation theorem and the orthogonal decomposition technique [47].

Let G be a graph built on samples $X^{\phi }=\left[\phi \left(x_{1}\right),\cdots ,\phi \left(x_{N}\right)\right]$ and A be a symmetric matrix that encodes the weighted adjacency information among images, that is,

(8) $$A_{ij}=\left\{\begin{array}{cc} \frac{1}{D_{i.}}\exp\left(-\frac{||\phi \left(x_{i}\right)-\phi \left(x_{j}\right){\left|\right|}^{2}}{\sigma_{i}^{\left(t\right)}\sigma_{j}^{\left(t\right)}}\right), & \;\phi \left(x_{i}\right)\in N_{K}\left(\phi \left(x_{j}\right)\right)\;\mathrm{or}\;\;\\ & \phi \left(x_{j}\right)\in N_{K}\left(x_{i}\right);\\ 0, & \;\mathrm{other}, \end{array}\right.$$

where $D_{i.}=\sum_{j}A_{ij}$ normalizes each weight and $\sigma_{i}^{\left(t\right)}$ (or $\sigma_{j}^{\left(t\right)}$) is the distance of $\phi (x_{i})$ (or $\phi (x_{j})$) and its *t*-th within-class neighbor, in our experiments, t=7. This settlement is more controllable than the traditional selection (e.g., the variance or predefined fixed value). By introducing the kernel function, this weight is rewritten as

(9) $$A_{ij}^{\phi }=\frac{1}{D_{i.}^{\phi }}\exp\left(-\frac{K_{ii}+K_{jj}-2K_{ij}}{\sqrt{\left(K_{ii}+K_{i^{\left(t\right)}i^{\left(t\right)}}-2K_{ii^{\left(t\right)}}\right)\left(K_{jj}+K_{j^{\left(t\right)}j^{\left(t\right)}}-2K_{jj^{\left(t\right)}}\right)}}\right),$$

if $\phi \left(x_{i}\right)\in N_{K}\left(\phi \left(x_{j}\right)\right)\;\mathrm{or}\;\;\phi \left(x_{j}\right)\in N_{K}\left(\phi \left(x_{i}\right)\right)$ and $A_{i,j}^{\phi }=0$ otherwise, where $i^{\left(t\right)}$ (or $j^{\left(t\right)}$) denotes the subscript of the *t*-th within-class neighbor of $\phi (x_{i})$ (or $\phi (x_{j})$). $D_{i.}^{\phi }=\sum_{j}A_{ij}^{\phi }$ is a normalizer. Based on this, we want to model the local intrinsic geometry structures and define a within-class neighborhood preserving scatter matrix in KPCA feature space as

$$\begin{array}{cc} S_{w}^{\phi } & =\sum_{k=1}^{2}\sum_{x_{i}\in C_{k}}\left(\phi {\left(x_{i}\right)}_{kpca}-\sum_{j=1}^{N_{k}}A_{ij}^{\phi }\phi {\left(x_{j}\right)}_{kpca}\right){\left(\phi {\left(x_{i}\right)}_{kpca}-\sum_{j=1}^{N_{k}}A_{ij}^{\phi }\phi {\left(x_{j}\right)}_{kpca}\right)}^{T}\\ & =\sum_{k=1}^{2}W_{kpca}^{T}K_{k}{\left(I_{k}-A_{k}^{\phi }\right)}^{T}\left(I_{k}-A_{k}^{\phi }\right){\left(K_{k}\right)}^{T}W_{kpca}\\ & =W_{kpca}^{T}K{\left(I-A^{\phi }\right)}^{T}\left(I-A^{\phi }\right)K^{T}W_{kpca} \end{array}$$

where $K_{k}$ is N×N kernel matrix whose first $N_{k}$ columns and first $N_{k}$ rows based block are taken from K and the rest parts are zero. $I_{k}$ is a $N_{k}\times N_{k}$ diagonal matrix. ${\left(I_{k}-A_{k}^{\phi }\right)}^{T}\left(I_{k}-A_{k}^{\phi }\right)$ preserves locality of nearby points with same class label in the embedding space if they are close in original space during the unfolding process of nonlinear structures.

In this context, minimizing a objective $w^{T}S_{w}^{\phi }w$ means to find a w that keeps the local geometry of within-class data as much as possible, so we integrate it to the *C*-SVM and define the primal problem as

(10) $$\min_{w,b}\left(\frac{1}{2}w^{T}w+C\sum_{i=1}^{N}L_{c}\left(x_{i}\right)+\frac{\eta }{2N}w^{T}S_{w}^{\phi }w\right),$$

where hinge loss $L_{c}\left(x_{i}\right)=\max\left(0,1-y_{i}\left(w^{T}\phi {\left(x_{i}\right)}_{kpca}+b\right)\right)$, C≥0, and η≥0 stands for a trade-off parameter to balance the penalties of within-class neighborhood preserving and maximum margin of the decision hyperplane in *C*-SVM. If η=0, the model will degrade to *C*-SVM where the within-class neighborhood preserved regularization does not work anymore.

Problem (10) is equivalent to the following formulation

(11) $$\min_{\bar{w},b}\left(\frac{1}{2}{\bar{w}}^{T}\bar{w}+C\sum_{i=1}^{N}L_{c}\left({\bar{x}}_{i}\right)\right),$$

where $\bar{w}=S^{1/2}w$, ${\bar{x}}_{i}=S^{-1/2}W_{kpca}^{T}K\left(x_{i},\cdot \right)$ and $S=I+\eta /2NS_{w}^{\phi }$. Thus it can be solved in standard *C*-SVM framework. We can simplify these computations via SVD (Singular Value Decomposition) technique to obtain $S^{\pm 1/2}$ because S is a real symmetric matrix [48]. Assume the optimal result of problem (10) is $\left(\alpha^{*},w^{*},b^{*}\right)$, the decision hyperplane becomes

(12) $$g\left(x\right)=sgn\left(\sum_{i=1}^{N}\alpha_{i}^{*}y_{i}\left(K{\left(x_{i},:\right)}^{T}W_{kpca}S^{-1}W_{kpca}^{T}K\left(x,:\right)\right)+b^{*}\right).$$

### 2.5. MIMO Within-Class Neighborhood Preserved ε-SVR Bounding Box Regression

Once the samples are classified as positive class using within-class neighborhood preserved *C*-SVC, they can be regarded as myocardium. However, their positions will not be completely overlapped with the true myocardium, due to the error of supervoxel merging and the classifier performance. To alleviate this side effect, we adopt bounding box regression technique to refine the proposal box.

Suppose (x,y,w,h) indicate the horizontal and vertical center coordinates of a detection box and its width and height, respectively, $x_{a}$ and $x_{p}$ are the center coordinates *x* of a detected box (called anchor) and a refined box (called proposal), respectively (the same applies to *y*, *w* and *h*), then a bounding box regression vector $z\in R^{4}$ could be presented by parameterizing the transformation between the anchor and the proposal (that is, the bounding box that seems to enclose a true myocardium), i.e., $z_{1}=\left(x_{p}-x_{a}\right)/w_{a}$, $z_{2}=\left(y_{p}-y_{a}\right)/h_{a}$, $z_{3}=w_{p}/w_{a}$, $z_{4}=h_{p}/h_{a}$. When z is learned from the samples and the ground truth, the anchor can be transformed into a refined proposal box. In this paper, we regard it as a multiple input multiple output (MIMO) regression problem and propose a MIMO within-class neighborhood preserved ε-support vector regression (SVR) to solve it. Different from the linear regression in RCNN [8] and Faster RCNN [9,10], the multidimensional regression will help to exploit the dependencies in the channel and will make each estimate less vulnerable to the added noise. Treating all the channel paths together will allow to accurately estimate each of them when only scarce data is available.

The traditional solution of MIMO problem is splitting multi-dimensional output into multiple single-dimensional outputs, which means constructing an independent regression model for each output dimension. Although this kind of method has simple implementation, it is computationally expensive and incapable of containing useful information among outputs. Another solution is multivariate statistical regression. However, this kind of method is sensitive to the changes of data so that it cannot be applied broadly. In present machine learning techniques, artificial neural network is the most common method to establish MIMO model. However, when facing small-scale sample problem, this method easily falls into local minimum and leads to over-fitting [49,50].

Suppose the elements in ${\left\{x_{i},z_{i}\right\}}_{i=1}^{N}$ denote SSAE learned samples and their corresponding multiple output, our MIMO ε-SVR approach defines the problem

(13) $$\min_{T,b}\left(\frac{1}{2}\sum_{j=1}^{4}\left|\right|t^{j}{\left|\right|}^{2}+C\sum_{i=1}^{N}L_{r}\left(u_{i}\right)\right),$$

where $T=[t^{1},\cdots ,t^{4}]$, $b={\left[b_{1},\cdots ,b_{4}\right]}^{T}$, $e_{i}=z_{i}-T^{T}\phi {\left(x_{i}\right)}_{kpca}-b$, $u_{i}=\sqrt{e_{i}^{T}e_{i}}$, the loss function is $L_{r}\left(u_{i}\right)={\left(u_{i}-\varepsilon \right)}^{2}$ when $u_{i}\geq \varepsilon$ and 0 otherwise. We follow the nonlinear mapping in the previous subsection as $\phi {\left(x_{i}\right)}_{kpca}=S^{-1/2}W_{kpca}^{T}K\left(x_{i},\cdot \right)$ and $S=I+\eta /2NS_{w}^{\phi }$.

By adopting the cost function $L_{r}\left(\cdot \right)$, MIMO ϵ-SVR is capable of finding the dependencies between outputs, and can take advantage of the information of all outputs to get a robust solution. As problem (13) cannot be solved straightforwardly, an iterative method was proposed to obtain a desired solution. By introducing a first-order Taylor expansion of cost function $L_{r}\left(\cdot \right)$, the objective of problem (13) will be approximated by the following objective

(14) $$\min_{T,b}\left(\frac{1}{2}\sum_{j=1}^{4}\left|\right|t^{j}{\left|\right|}^{2}+C\sum_{i=1}^{N}a_{i}u_{i}^{2}+const\right),$$

where $a_{i}=2\gamma \left(1-\varepsilon /u_{i}^{k}\right)$ when $u_{i}^{k}\geq \varepsilon$ or 0 otherwise, const is constant term which does not depend on T and b, and the superscript *k* denotes *k*-th iteration. According to the Representer Theorem, the best solution of minimization of problem (14) in feature space can be expressed as $t^{j}=\sum_{i}\phi {\left(x_{i}\right)}_{kpca}\beta^{j}$, then the linear search algorithm can be readily expressed in terms of $\beta^{j}$. In fact, our model can be solved using the standard approach [49,50], just need to replace the mapping by our $\phi {\left(x_{i}\right)}_{kpca}$.

Once $\beta^{j}$ has been computed, for a new SSAE-learned vector $x_{i}$ with $\left(x_{a},y_{a},w_{a},h_{a}\right)$, we can estimate the *j*-th output as

(15) $${\hat{z}}^{j}\left(x_{i}\right)=\left(\sum_{i=1}^{N}\left(K{\left(x_{i},:\right)}^{T}W_{kpca}S^{-1}W_{kpca}^{T}K\left(x,:\right)\right)\beta^{j}\right),$$

so the position of the corresponding refined proposal is $\left(x_{a}+w_{a}{\hat{z}}^{1},y_{a}+h_{a}{\hat{z}}^{2},w_{a}{\hat{z}}^{3},h_{a}{\hat{z}}^{4}\right)$. It is noted that this nonlinear regression technique is not conducted on all of the supervoxel regions, but just regions near to the ground truth by using intersection-over-union to measure their overlapping (e.g., larger than 0.6). Thus, this step can be regarded as the closing step of refinement.

### 2.6. Refinement

There is a sample imbalance problem in our detection model. Specifically, for each image in the training set, the intersection-over-union (IoU) index can be computed between the labeled location of the target and generated location by the model: $IoU=\frac{GTB\cap DB}{GTB\cup DB}$, where GTB and DB denote the ground truth of the target and detection result respectively. When IoU approaches to 1, the detection result overlaps the ground truth in more parts and vice versa. Generally, when constructing a training set, a region is regarded as positive sample if its IoU exceeds a threshold (e.g., 0.5), and negative if IoU is less than another threshold (e.g., 0.3). But the number of negative samples in the training set is practically much larger than the positive samples because the parts occupied by the target on an image are often much smaller than those of the non-target. This imbalance has a remarkable impact on the training of the model and results in a false positive problem where many negative samples are misclassified as positive samples.

We employ the hard negative mining [24] strategy to solve this problem. After the deep model and the classifier are trained, the training set is sent to the model, and the classifier gives the probability that each region belongs to the positive sample. Then we pick out the samples with high scores and misclassified into positive samples and called them as “hard to share negative samples”. From them we chooses those samples that are not only misclassified but also less than 0.1 in IoU as difficult negative samples, then feed them into the pre-trained model and conduct iterative fine-tuning. By this way, the training samples are balanced to improve the model’s performance.

Furthermore, for each candidate region, the proposed *C*-SVM classifier will output its probability of each class. There are usually many candidates around the true myocardium region, so the final detected area should be inferred from these regions. In our experiments, we employ the non-maximum suppression (NMS) algorithm [51] to eliminate redundant (cross-repeated) candidates and find the best myocardium position. Since the overlap between the candidates is sometimes relatively large, it is necessary to remove these regions with higher IoU scores (e.g., larger than 0.3) between the overlapped area. Usually, only a small number of the most likely areas are remained after this processing.

## 3. Datasets and Evaluation Metrics

### 3.1. Cardiac MRI Dataset and Preprocessing

The heart MRI data used in our experiments are from the publicly available Cardiac Atlas Project (CAP) data set, a collaborative database created by multiple organizations [52]. The data set contains 83 patients with short axis cardiac MRI images, where the MRI scanners used to collect these images include Siemens (Avanto 1.5T, Espree 1.5T and Symphony 1.5T), Philips (Achieva 1.5T, 3.0T and Intera 1.5T), and GE (Signa 1.5T). Because of the different characters of acquisition equipments, the parameters of the heart MRI images in different patients are varying. The formation of the images were in presence of remarkable offset or distortion. The parameters of the typical short-axis cardiac MRI images are: thickness 6 mm, gap 4 mm or thickness 8 mm, gap 2 mm. The size of these images is between 192×156 and 512×512, and the resolution of each voxel is between 0.7×0.7×6 and 2.0×2.0×10 (in mm). The number of slices per patient image sequence is 8∼17, and each slice corresponds to approximately 18∼35 images (one cardiac cycle).

In spite of these varying changes across subjects and acquisition equipments, we took myocardium as a preprocessing of the subsequent steps such as registration or segmentation, so we did not adjust these images into a common image space with same spatial resolution. We only rearranged their orientation as RAI automatically and employed the N4 regularization algorithm [53] to correct the deviation field of each heart MRI image.

The myocardium region in each image was manually labeled by two well-trained students. Since this labeling was a non-trivial and challenging problem due to the projective nature of the data, fuzzy organ boundaries, and large anatomical variability, their results were carefully cross-checked and further checked by a radiologist to made a final result as gold standard for evaluation. For each subject, this processing took about 2 h.

### 3.2. Evaluation Metrics

Considering the limited size of the data set, we randomly divided 83 cardiac image sequences into three folds of approximately equal size (28, 28, and 27 subjects) for training, validation, and test. The three-fold cross-validation was taken as the evaluation prototype, that is, our experiments were conducted three rounds, in each round we trained our model in the training set, adjusted the hyper-parameters in the validation set, and applied the model in the remaining test set for measuring its performance.

We employed true positive rate (or Tpr, sensitivity, Se, recall), positive predictive value (or Ppv, precision), F1, and area under the receiver operating characteristic curve (AUC) to measure the performance of the proposed method. Tpr (resp. specificity) is a measure of effectiveness in identifying regions with positive (resp. negative) classifications. Specifically, the chosen metrics are defined as Tpr=tp/(tp+fn) and Ppv=tp/(tp+fp), where tp, tn, fp and fn indicate the true positive (correctly identified regions), true negative (correctly identified background regions), false positive (incorrectly identified regions), and false negative (incorrectly identified background regions), respectively, and all the pixels are equally treated towards their bounding box without considering the tissue they depict. F1 is a harmonic mean of the precision and recall measures and can be used to measure the degree of similarity of the two sets. The expression is expressed by the following equation F1=2Tpr×Ppv/(Tpr+Ppv). The value of F1 is between [0–1] and the larger the value of F1, the more similar the two sets. DR=2(|A∩B|)/(|A∪B|), where *A* is the ground truth region, *B* indicates the detected region, and |A∩B| and |A∪B| denote the number of pixels in the intersected region and in the union region, respectively. All of accuracy and AUC and DR measure the overall detection performance.

Statistical analysis is performed as appropriate in order to evaluate the relative performance of different detection methods. Due to the relatively small number of images, p<0.05 is considered to be statistically significant. All the experiments were carried out in MATLAB2016a with deep learning toolbox and parallel computing toolbox on a PC with an Intel Core i7-3770K CPU, 3.70GHz, GTX1070 GPU, and 16GB RAM. Since the negative samples are remarkably more than the positive ones, total feeding all samples into the SSAE network will make the model biased. To alleviate this, a batch training strategy was adopted.

## 4. Experimental Results and Discussion

The proposed detection method was evaluated from two aspects: effectiveness of the parts in the model, and the comparison with close related state-of-the-art detection methods. Also an experimental investigation was carried out in the next section on the parameter setting, i.e., the number of supervoxels, the size of proposed region, the structure of the SSAE network and the *C* and β in our SVM classifier and regressor.

### 4.1. Parameters Setting

#### 4.1.1. Number of Supervoxels and Training Image Set Building

**Figure 4:**
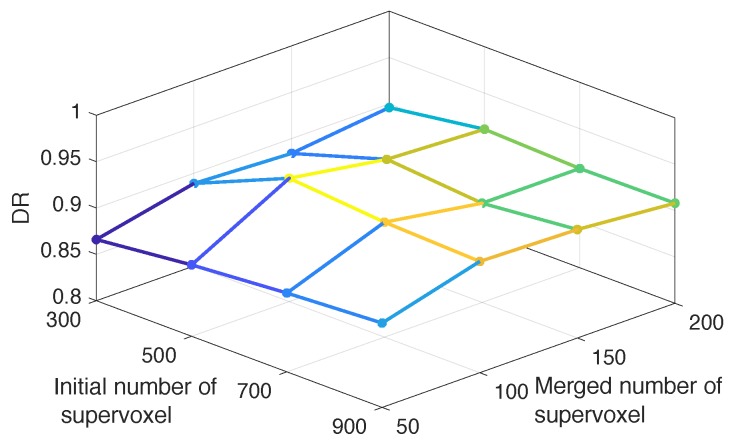
Mean Dice ratio on the training set using four different *M* and *N* values.

There are two parameters (the number of initial supervoxels *M* and the final number of merged supervoxels *K*) in the candidate region generation module. Since we intend to make the true blood pool of left ventricle locate in the merged supervoxels, we employed the Dice ratio (DR) index to measure the similarity between the true object and the supervoxel in the region of this object under different *M* and *K*. The search range of the initial supervoxel is {300,500,700,900} and that of the merged supervoxel is {50,100,150,200}. Figure 4 presents the DR values corresponding to different parameters and it is shown that when the initial number is greater than 500 and merged number is greater than 100, the DR values are acceptable. The maximum DR reaches for M=500 and K=100, so we choose them as the parameters in the following experiments.

Once the merged number was settled, the positive samples were constructed as the bounding regions of the merged supervoxel that located in the real region of the left ventricle target on each training image; while the negative samples were those rest regions.

#### 4.1.2. Size of Proposed Region and SSAE Training

The region proposals were scaled to a fixed size (i.e., τ×τ) and sent to SSAE network for feature learning. The value of τ was determined through cross-validation to make the number of neurons in the SSAE input layer, specifically, the number of neuron in each encoding layer was hoped much less than that of the input neurons for achieving the sparse encoding, so we tested multiple values of τ in four kinds of three-hidden layer SSAEs with different numbers in each layer, i.e., {576,400,300,200} for τ=24; {1296,750,450,250} for τ=36; {2304,1150,600,300} for τ=48; and {3600,1600,750,350} for τ=60.

**Figure 5:**
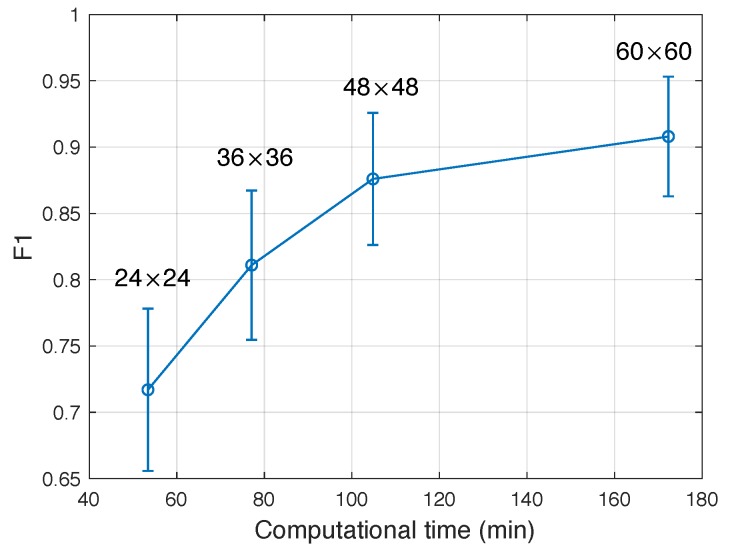
Detection accuracy (F1) on the verification set with model training time on the verification set using four different τ values.

The models of four different SSAE structures were trained to adjust the optimal performance of each model in different SSAE super-parameters, including weight penalty factor λ, sparse penalty coefficient β, and coefficient factor ρ. The values of the three super-parameters were settled as $\lambda \in \{10^{-1},10^{-2},10^{-3}\}$, β∈{1,0.6,0.3,0.1}, and ρ∈{0.01,0.05,0.1,0.2}. Figure 5 presents the detection accuracy (F1) versus training time of the test model under four different τ values on the verification set. It can be seen from the figure that the overall trend is the higher the value of τ, the higher the detection accuracy of the model, along with the longer the training time. When τ reaches 60, the model obtains best test accuracy, so τ is set to 60. The values of three super-parameters are set to $\lambda =10^{-2}$, β=0.3, and ρ=0.2.

#### 4.1.3. Parameters of Within-class Neighborhood Preserved *C*-SVC and ε-SVR

**Figure 6:**
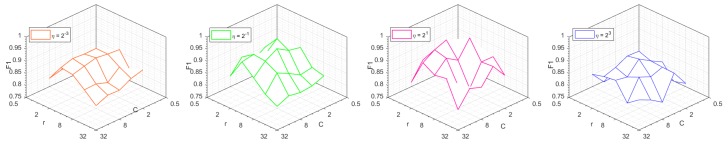
Detection accuracy (F1) on the verification set using different *r*, *C*, and η values.

Considering choosing the kernels and the parameters for the SVM-based methods is still an open problem, we adopted grid searching strategy to settle these parameters. The typical kernel used in our experiments is the Gaussian kernel, i.e., $\exp\left(-{\left(u-v\right)}^{T}\left(u-v\right)/2\sigma^{2}\right)$ or $\exp\left(-r{\left(u-v\right)}^{T}\left(u-v\right)\right)$, where σ controls the width of kernel while *r* is suitable for numerical searching. *r* should be larger than 0 in the sense of similarity. We selected *r* from $\left\{2^{-3},2^{-1},2^{1},2^{3},2^{5}\right\}$. For all *C*-SVM based methods, the common parameter is the slack variable *C*, we selected it from $\left\{2^{-3},2^{-1},2^{1},2^{3},2^{5}\right\}$; for the additional trade-off parameter η, we determined it from $\left\{2^{-3},2^{-1},2^{1},2^{3}\right\}$. To speed up the searching, the ranges were also restricted with respect to the data prior information. Figure 6 presents the average detection accuracies corresponding to different parameters. Overall speaking, smaller parameters are helpful for obtaining better accuracies, so we settle C=0.5, r=2, and η=2 in all experiments.

Similarly, for parameters of within-class neighborhood preserved ε-SVR, we still adopted grid searching strategy to settle the parameters *r* in Gaussian kernel, the slack variable *C*, and the additional trade-off parameter η from the above ranges, and the parameter ε that sets the width of insensitivity zone of the regressors cost function was chosen from {0.01,0.5,1.0,1.5,2.0}. According to the best F1 accuracy, we settle C=0.5, r=2, η=2, and ε=1.5 in all experiments.

### 4.2. Validation of the Parts in Our Model

There are four main components in our model: structural similarity-enhanced supervoxel over-segmentation; deep SSAE feature learning; within-class neighborhood preserving-induced *C*-SVC and MIMO ε-SVR. To verify their roles in our model, we replaced each component into a state-of-the-art model and built five compared methods: (1) our model with SLIC that replaces our supervoxel over-segmentation; (2) our model with intensity feature that replaces SSAE learned feature; (3) our model with Softmax classifier that replaces the proposed classifier; (4) our model with *C*-SVM that replaces the proposed classifier; (5) our model with linear regression [8,9] that replaces the proposed regressor.

**Figure 7:**
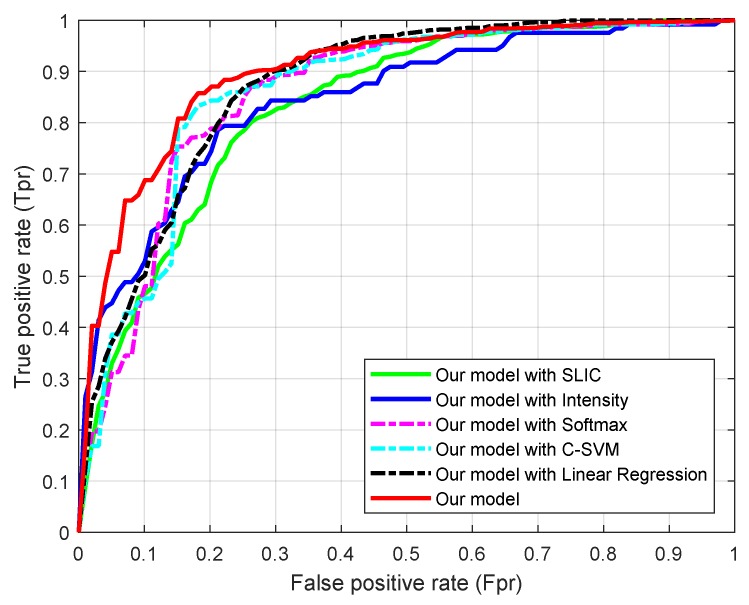
Receiver operating characteristics (ROC) curves of the proposed method with different modules.

In order to objectively measure the performance of these five variations, the false positive rate and true positive rate of the detection results derived by different versions were calculated, by sweeping a threshold from 0 to 1 over the final classification output. The averaged results over them are plotted as receiver operating characteristics (ROC) curves in Figure 7. It is seen that our method with these components achieves the best performance. Also, when the within-class neighborhood preserved *C*-SVC is replaced by the standard *C*-SVM, the performance are better than those by the Softmax classifier. Furthermore, the over-segmentation and feature extraction methods are also essential for improving the overall accuracy. If the supervoxels were generated with SLIC, or only the intensity feature was adopted, the ROC curves increase much slower than others.

Table 1 shows the performances of different versions in detecting the myocardium. It shows that the proposed method achieves competitive results: the mean F1,Tpr,Ppv, and AUC are 0.924, 0.936, 0.916, and 0.891, respectively, remarkably higher than the proposed method with other modules. Statistical analysis shows that the performance of the proposed method is significantly higher with the SLIC, intensity, Softmax, and linear regression versions (in the level p<0.05).

**Figure 8:**
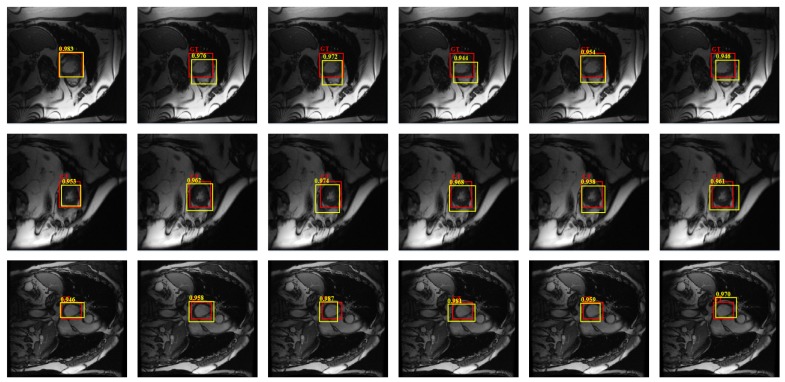
Detection results of our model with different parts, where red rectangles denote ground-truth and yellow ones denote results by compared methods. Each row from left to right: Our model; our model with SLIC; our model with intensity; our model with Softmax; our model with *C*-SVM; our model with linear regression, respectively.

Figure 8 shows the detection results of different versions on three randomly selected cardiac MRI images in the test set, where red rectangles denote ground-truth and yellow ones denote results by compared methods. The larger overlapping means the corresponding method is better. As can be seen from the figure, our overall detection model is more robust, and the detection model based on *C*-SVM also achieves competitive results, which also demonstrates the strong ability of SSAE to learn the deep feature for classification, it can effectively distinguish different category of samples, thereby reducing the requirements of the follow-up classifiers.

### 4.3. Comparison with Related Methods

In this subsection, we carried out a comparative study between the proposed method and the state-of-the-art ones for the detection of myocardium over the cardiac datasets. Since the detection is based on the framework of region proposal and classification, such comparative study will help further explain its characteristic reported in the last section. To this end, five representative detection methods were selected: enhanced cascade detector (BCD) [6], RCNN [8], Faster RCNN [9], YOLOv3 [10,21], and a single-shot refinement neural network (RefineDet) [23].

BCD [6] is a well-known detection algorithm that was originally used in face detection and it trains the cascade AdaBoost to classify the regions that are generated by sliding windows and represented by Haar-like features. We took it as a representative of traditional detection methods.

RCNN [8] is a typical region proposal based convolutional neural network for object detection. This method firstly applies selective search method to generate around 2000 category-independent region proposals, then the features of each region proposal are extracted by a pre-trained convolutional model, finally the top-level features are classified by linear SVM. Faster RCNN [9] brings major improvements to traditional CNN by designing a region proposal network that extracts candidate areas instead of wasting time on selective search, which significantly accelerates the detection. On the other side, YOLOv3 [21] is an improved version of the state-of-the-art, real-time YOLOv2 [10] that applies a single neural network to the full image. The network divides the image into regions and predicts bounding boxes and probabilities for each region. These bounding boxes are weighted by the predicted probabilities. We take it as a representative of region proposal independent neural network detection method. RefineDet [23] is a recent single-shot based detector that consists of the anchor refinement module and object detection module to achieve better accuracy than two-stage methods and maintains comparable efficiency of one-stage methods. We took the RefineDet320+ and VGG-16 net as the training model.

**Figure 9:**
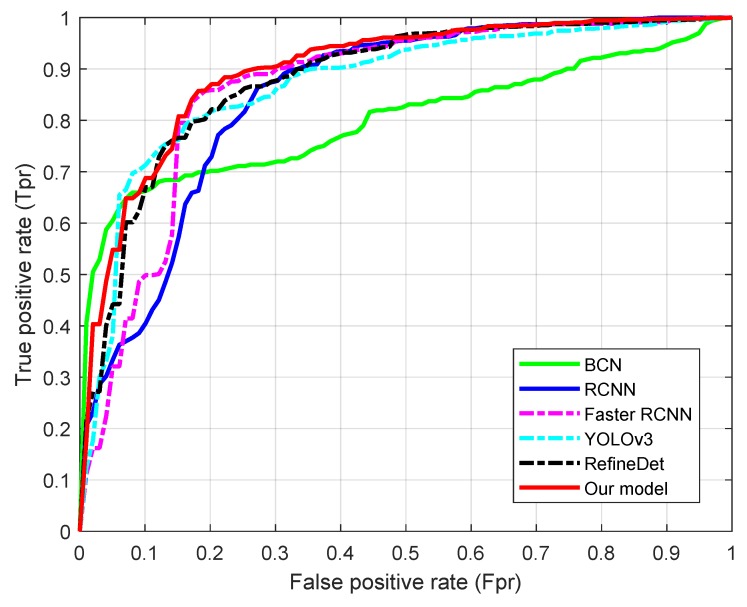
Receiver operating characteristics (ROC) curves of the compared methods.

The average results over the compared methods are plotted as receiver operating characteristics (ROC) curves in Figure 9. It can be seen that our method consistently outperforms its competitors RCNN and Faster RCNN and it is also competitive to recent advancements YOLOv3 and RefineDet. Overall it achieves the best performance, while BCD is the worst among these methods. Furthermore, Faster RCNN performs similar to our method and outperforms RCNN.

The average accuracies of the detection based on the six methods are shown in Table 2. It can be seen from the table that the accuracy of the proposed algorithm is superior to that of the other five algorithms, the worst performance is BCD and Faster RCNN obtains the best accuracy among the convolutional neural network based methods.

**Figure 10:**
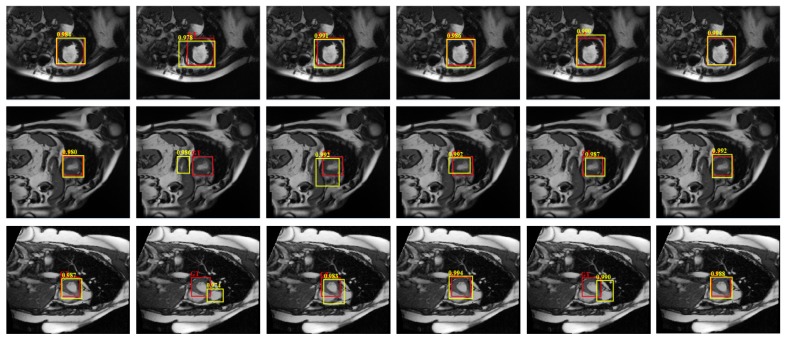
Examples of detection results, where red rectangles denote ground-truth and yellow ones denote results by compared methods. Each row from left to right: Our model; BCD; RCNN; Faster RCNN; YOLOv3, and RefineDet, respectively.

Figure 10 shows the detection results of three randomly selected images in the testing set. In each row, red rectangles denote ground-truth and yellow ones denote results by compared methods. The values on the top of the bounding box is the maximal probability outputs by each method. The results show that our method can detect various myocardium with high quality, in most cases, the overlap between the detection of the final results and the target real area is higher, which is benefited from the combined candidate region generation, classification and regression algorithm. Faster RCNN and RefineDet also performs well in these images, except some small difference in overlapping. YOLOv3 also locates the accurate object in most images, but was disturbed by complicated surrounding tissues. In comparison, BCD is the worst detector and most resulting locations are low quality.

### 4.4. Discussion

#### 4.4.1. Detection Performance

With regard to myocardium detection performance, the F1 and AUC measures indicate our method achieves a higher performance than previous studies using hand-crafted features such as the HOG or intensity in BCD method. They also tell that it is necessary to design a specific detection model towards a specific medical object. As we know, RCNN, Faster RCNN, YOLOv3, and RefineDet are state-of-the-arts in object detection, however, they are designed for general multiple objects detection and their advantages aren’t thoroughly embodied for myocardium detection in our task. The F1,Tpr,Ppv,AUC measures of our method are higher 1.0%, 1.8%, 1.8%, 1.6% than the best of these methods (RefineDet). Statistical analysis show that these four metrics of our method significantly outperforms Faster RCNN (in the level p<0.05). The AUC and Ppv of our model significantly outperforms Fast YOLOv3 and RefineDet (in the level p<0.05).

Our results indicate the strong classifiers such as *C*-SVM are helpful for achieving higher performance than Softmax. In our proposed *C*-SVM classifier detection model, SSAE and *C*-SVM training were carried out independently, that is, we first conducted SSAE unsupervised training, and then used the learned features to train the proposed *C*-SVM classifier. According to Softmax classifier, SSAE and Softmax training can be combined to form a whole part, that is, we first conducted SSAE unsupervised pre-training, and then connected SSAE and Softmax. The classification error of Softmax can be propagated back to SSAE, and the detection effect based on Softmax classifier is logically consistent. However, as pointed by RCNN [8], Softmax does not outperform SVM for classifying objects. Our experimental results also support this conclusion. Nevertheless, it is necessary to use the loss function of SVM to design error back propagation approach to the SSAE network in our next work.

With regard to region detection, although the myocardium is an ellipsoid-like tissue, the two-dimensional image of the myocardium varies in size and shape due to variations in motion and acquisition. For this reason, using only a single fixed-sized window is insufficient to detect myocardium regions with various shapes. Although a fixed-sized window has been used in RCNN, the detected myocardium is resized by a fixed-sized bounding box, regardless of its shape. On the other hand, our approach similarly feeds a fixed-sized window to SSAE network; however, inside SSAE, varying bounding boxes are possibly more suitable for the shape of myocardium by scanning the input image with multiple supervoxels with different scales and aspect ratios. To make use of SSAE, we set a slightly larger window to include multiple myocardium and background in the four corners. This was not optimal because SSAE learns the region of myocardium and background at the same time, and it is better to exclude various backgrounds in an input image to learn positive regions. This will be further investigated in our future work.

Furthermore, the structure prior information hidden in medical images is useful for accurate myocardium detection. For example, we proposed the structure similarity induced supervoxel instead of simple intensity similarity based supervoxel; we also proposed to incorporate the within-class neighborhood preserved scatter matrix to standard *C*-SVM classifier, which remarkably improves the overall performance of our model. As we can find from the first experiment that evaluated the major parts of our model, the consideration of structure prior information enhanced the distinctiveness of myocardium object from the complex background.

**Figure 11:**
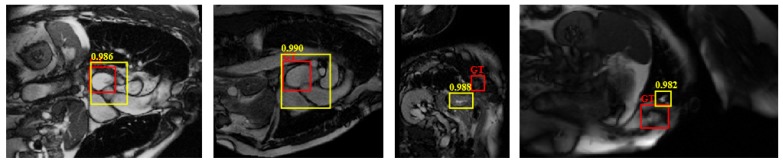
Examples of failure detection results by our method, where red rectangles denote ground-truth and yellow ones denote our results.

The detection model provides a good detection effect for most of the heart MRI images in the CAP data set, but there are still some failures in our experiments. Figure 11 presents some error detection. For the sake of comparison, two images of each column in the figure come from the same patient’s heart MRI image sequence, which shows that the place where false detection occurred. The errors mainly appear at the head and tail of the heart image sequence. At these places, the sizes of the left ventricle tissue are very small, the shapes are not obvious, and the right ventricle occurred with more adhesion to left ventricle. When the left ventricle with the normal form (as shown in the first line), the accuracy of outer frame is almost the same as that of the real bounding box.

#### 4.4.2. Processing Speed

The four parts, i.e., the supervoxel-based region proposal, the SSAE feature learning, and the within-class neighborhood preserved *C*-SVM, and ε-SVR, in our model took different time in training. Compared with thirteen minutes to train the SVM model and one minute to generate region proposals, the SSAE took much longer time and it took about three hours. Because our approach feeds relatively large-size images to SSAE, it requires the number of mini-batches to be reduced to one because of the size of the GPU memory, leading to a slow learning rate for stable learning. Therefore, it is necessary to increase the number of iterations to train the network sufficiently. Fortunately, our SSAE is not a deeper structure that contributes to the long training time, like the VGG-16 in some CNN based models. Once the training is finished, our model averagely took less than one minute to extract the myocardium from a cardiac image with the trained model. This performance seems to be comparable in medical applications. This high-throughput approach may have some advantages in practical usage in hospitals and laboratories to assist pathologists in their daily tasks.

#### 4.4.3. Limitations of the Work

There are several limitations that need to be addressed. Firstly, this work, like most neural network-based cardiac MR image analysis studies [4,29,30], suffers from the restriction of available ground truth data to a limited number of cardiovascular disease diagnoses, such as pulmonary hypertension, congenital heart disease, coronary heart disease, and dysplasia. Therefore, results of this study can only show performance on limited set of patients. The number ot layers in SSAE learning was also restricted. Besides, currently available data largely consists of short-axis image where boundaries between the blood pool and myocardium are more or less clearly visible. More challenging image such as long-axis image with large spatial interval sampling are not part of the current sets and should be researched on in future work.

More importantly, this study focused on the relation of left ventricle and its myocardium, and didn’t consider the relation of other cardiac structures (such as right ventricle and its myocardium, left atrium, right atrium). In fact, these structures have strong anatomical prior that can guide the accurate localization of myocardium and left ventricle. Future studies may gain insight from the recent advance on the CNN-based relation modeling among detected objects [18], the CNN-based iterative localization refinement [19], and explore the multiple objects detection by extending the proposed framework.

## 5. Conclusions

In cardiac MR image analysis, left ventricle and myocardium detection is often used as a prerequisite step, which plays a key role in the successive steps such as image registration and segmentation. This paper has presented a new efficient detection approach to myocardium structures in cardiac MR images through an enhanced region proposal-based model. The model first proposed a structural similarity-enhanced supervoxel over-segmentation and hierarchical clustering approach to extract candidate regions; then, the deep features were learned by SSAE network; furthermore, the learned features are classified by a within-class neighborhood preserved *C*-SVC, and during the refinement, the bounding boxes are adjusted by a multiple-intput multiple-output within-class neighborhood preserved ε-SVR regression and hard negative sample mining technique. Different parts in our model were also tested and prediction accuracies validated the advantage of proposed integration. Furthermore, comparative experiments demonstrated that the proposed model achieved a better detection accuracy on the publicly available dataset. The model does not require a large amount of training data and learns from coarsely annotated volumetric images (bounding-box masks). It can be potentially extended to similar object detection in other medical MR images.

## Figures and Tables

**Table 1:** Performances (average ± standard deviation) of variations of our method.

Metric	Proposed Method with All Terms	Proposed Method with SLIC	Proposed Method with Intensity Feature	Proposed Method with Softmax	Proposed Method with *C*-SVM	Proposed Method with Linear Regression
F1	0.924 ± 0.034	0.852 ± 0.036	0.861 ± 0.038	0.878 ± 0.042	0.904 ± 0.035	0.898 ± 0.044
Tpr	0.936 ± 0.037	0.867 ± 0.048	0.884 ± 0.042	0.894 ± 0.040	0.915 ± 0.036	0.890 ± 0.038
Ppv	0.916 ± 0.028	0.838 ± 0.032	0.847 ± 0.037	0.866 ± 0.042	0.894 ± 0.039	0.885 ± 0.046
Area under ROC (AUC)	0.891 ± 0.031	0.824 ± 0.026	0.838 ± 0.032	0.851 ± 0.024	0.857 ± 0.030	0.862 ± 0.033

**Table 2:** Performances (average ± standard deviation) of six compared methods.

Metric	Proposed Method	BCD	RCNN	Faster RCNN	YOLOv3	RefineDet
F1	0.924 ± 0.034	0.801 ± 0.092	0.870 ± 0.061	0.896 ± 0.058	0.878 ± 0.065	0.914 ± 0.046
Tpr	0.936 ± 0.037	0.805 ± 0.097	0.877 ± 0.062	0.908 ± 0.056	0.892 ± 0.062	0.918 ± 0.041
Ppv	0.916 ± 0.028	0.798 ± 0.103	0.863 ± 0.069	0.874 ± 0.062	0.862 ± 0.060	0.898 ± 0.045
Area under ROC (AUC)	0.891 ± 0.031	0.798 ± 0.026	0.858 ± 0.037	0.872 ± 0.025	0.870 ± 0.032	0.875 ± 0.036
